# Draft Genome Sequence of *Mycobacterium ulcerans* S4018 Isolated from a Patient with an Active Buruli Ulcer in Benin, Africa

**DOI:** 10.1128/genomeA.00248-17

**Published:** 2017-04-27

**Authors:** Stanimir Kambarev, Stéphane Corvec, Annick Chauty, Estelle Marion, Laurent Marsollier, Frédéric Pecorari

**Affiliations:** a CRCINA, Inserm, CNRS, Université d’Angers, Université de Nantes, Nantes, France; b CRCINA, Inserm, Université d’Angers, Université de Nantes, Nantes, France; c Service de Bactériologie et Hygiène Hospitalière, CHU de Nantes, Nantes, France; d CDTUB de Pobè, Pobè, Benin; e CRCINA, Inserm, Université de Nantes, Université d’Angers, Angers, France

## Abstract

Currently, there are only two publicly available genomes of *Mycobacterium ulcerans*—the causative agent of the neglected, but devastating, tropical disease Buruli ulcer. Here, we report the draft genome sequence of isolate S4018, recovered from an active cutaneous lesion of a patient with Buruli ulcer in Benin, Africa.

## GENOME ANNOUNCEMENT

The neglected, but devastating, tropical disease Buruli ulcer is the third most common mycobacteriosis worldwide after tuberculosis and leprosy. It is associated with extensive subcutaneous necrosis caused by the human pathogen *Mycobacterium ulcerans* (1). Surprisingly, since the sequencing of the first genome of this species in 2007 (2), there has been only one other genome submitted to the public nucleotide databases (draft sequence, strain Harvey, accession number JAOL01000000). Here, we report the draft genome of isolate S4018, recovered from a cutaneous lesion of patient with Buruli ulcer in Benin, Africa.

The isolate was cultivated on Lowenstein-Jensen medium for 5 months. Prior to DNA extraction, it was grown on Middlebrook 7H10 agar and enriched with an OADC (oleic acid, albumin, dextrose, and catalase) supplement. Cells were then scraped from the agar and washed with wash solution (0.3 M sucrose, 50 mM Tris pH 8.0, and 10 mM EDTA). Every 50 mg of cells were treated with 3.5 mM lysozyme and 0.4 mM RNase A in 20-µL volume overnight at 37°C, followed by centrifugation. The pellets were resuspended in 1 × Tris/EDTA (TE) buffer, containing 36 nM proteinase K and 1% SDS. After incubation for 1 h at 50°C and another centrifugation, the pellets were resuspended in lysis solution (6% guanidine hydrochloride, 1% Tween 20, and 1% Nonidet P-40) and incubated for 1 h at 37°C. Finally, the cells were completely lysed with a bead beater, and the aqueous phase of the lysates was extracted with chloroform. DNA was precipitated with isopropanol/3 M sodium acetate on ice. The precipitates were washed with 70% ethanol, dried, and dissolved in 1 × TE buffer. DNA was fragmented (200 to 300 bp) using a Bioruptor Standard (Diagenode) device. Sequencing libraries were prepared from 1 µg of fragments using a NEBNext Ultra DNA library prep kit for Illumina (NEB) and sequenced on a MiSeq sequencer (Illumina). *De novo* assembly was performed with Velvet version 1/2/10 (3) and VelvetOptimiser version 2.2.5 (4) from 4,059,460 high-quality paired-ends reads (150 bp). The obtained contigs were reordered with Mauve version 1/2/10 (5) against the complete genome of strain Agy99 (2). Finally, the assembly was annotated through the NCBI Prokaryotic Genome Annotation Pipeline (6).

The draft sequence consists of 503 contigs with an average coverage of 39× and an *N*_50_ of 37 kb. It has a G+C content of 66% and a total length of 5,399,220 bp. Annotation revealed 4,914 coding sequences, 1,035 pseudogenes, and 51 RNA genes. We attributed the high number of contigs to the characteristic accumulation of the IS*2404* and IS*2606* mobile elements in the genome of *M. ulcerans* (2, 7), since it has been shown that such repeated elements may interfere with the sequence contiguity of assemblies from short reads (<1,000 bp) (8, 9). Therefore, even though Illumina is one of the most established platforms, more recent “third-generation” approaches performing at longer read lengths, such as the approach with the PacBio platform (10), might be more suitable for sequencing *M. ulcerans* genomes.

### Accession number(s).

The draft genome of *M. ulcerans* S4018 sequenced under this project has been deposited at DDBJ/EMBL/GenBank under the accession number MDUB00000000. The version described in this paper is the first version, MDUB01000000.
